# Association of Fracture Location and Pattern With Nonunion or Malunion in Tibia Fractures Managed With Intramedullary Nailing: A Retrospective Study

**DOI:** 10.7759/cureus.49156

**Published:** 2023-11-21

**Authors:** Mahmood A Alam, Ahmed F Shirazi, Hasan Alaradi

**Affiliations:** 1 Orthopaedics and Trauma, Salmaniya Medical Complex, Manama, BHR

**Keywords:** tibia nail, tibia shaft fracture, nonunion of tibia, malunion of tibia, intramedullay nailing of tibia

## Abstract

Background and objective

Extra-articular fractures of the tibia are common orthopedic injuries that are frequently treated with rigid intramedullary nailing. Fracture location and pattern may increase the risk of nonunion or malunion in fractures managed with intramedullary nails. This study aimed to assess the relationship between fracture pattern and location with malunion and nonunion. The primary objective was to evaluate the influence of fracture location and pattern on adverse clinical outcomes such as nonunion, delayed union, and malunion in tibial shaft fractures that are treated operatively with rigid intramedullary nails.

Methodology

This was a retrospective cross-sectional study conducted on patients operated in a tertiary care center in the Kingdom of Bahrain. The study included patients who sustained tibia shaft fractures and were subsequently operated with intramedullary of the tibia. The primary endpoint was the rate of adverse outcomes associated with fracture patterns. Fracture characteristics were to determine which fracture patterns healed well with intramedullary nailing and which fractures ended up with malunion or nonunion and would likely benefit from additional measures to augment the nail fixation and help encourage union.

Results

One hundred and eighty-nine patients were included in the study. The level of associated fibula fracture was significantly associated with an increased risk of nonunion and malunion (*P *= 0.0034, *P* = 0.001). The presence of a concomitant distal fibula fracture in association with tibia fractures increased the odds of nonunion (odds ratio [OR] = 4.871, *P* = 0.033, confidence interval [CI] = 1.133-20.948).

Conclusions

The level and pattern of some tibia and fibula fractures were associated with nonunion, malunion, and delayed union. Further studies with more robust follow-up are needed to examine these findings in greater detail.

## Introduction

Extra-articular fractures of the tibia are common orthopedic injuries that account for about 2% of all adult fractures [1]. The incidence of these fractures was found to be around 16.9/100,000 in a recent large-scale study, with a bimodal distribution of peaks at ages 20 and 50 years [2]. Tibia fractures are especially prone to a prolonged healing course due to their long subcutaneous border and tenuous blood supply [3,4].

Tibial nonunion is a disabling condition and has detrimental effects on the quality of life of patients affected by it; the impact of tibia shaft nonunion on physical health was comparable to the reported impact of end-stage hip arthrosis and worse than that of congestive heart failure when looking at various mental and physical assessment scores [5]. Apart from the deleterious effect on patient outcomes, tibial nonunion has been found to significantly increase the financial burden on healthcare systems, with an estimated twofold increase in service costs when compared to patients without nonunion [6].

The significance of tibial malunion, on the other hand, is a subject of great debate, with most of the literature suggesting that corrections are reserved for patients who complain of pain, functional deficits, unacceptable cosmetic appearance, or an unacceptably high risk of degenerative osteoarthritis [7].

Various treatment modalities have been used to treat tibia fractures, such as casting, external fixators, plate fixation, and intramedullary fixation, with varying levels of success [8,9]. Rigid intramedullary nailing has emerged as the most commonly utilized modality of fixation for tibia shaft fractures, as it has been shown to be an effective and safe method of fixation for most fracture patterns [10]. Large-scale studies have established that nonunion rate may be associated with fracture location in various fractures [11]. Other studies have explored several factors that affect the union rate of tibia fractures [12,13].

Certain tibia fracture patterns and locations may require particular attention by orthopedic providers to avoid the adverse effects of nonunion and malunion. While some studies addressed factors that are associated with nonunion and reoperation rates in tibia fractures, this study focuses on the association of tibia fracture pattern with outcomes post rigid intramedullary nailing [14]. This paper aims to identify fracture patterns that are associated with a higher rate of nonunion, malunion, or delayed union.

## Materials and methods

The study was conducted at Salmaniya Medical Complex, a tertiary referral center and the prime trauma center in the Kingdom of Bahrain. The study retrospectively assessed all extra-articular tibia fractures treated with reamed rigid intramedullary nailing between January 2019 and May 2023. The follow-up period of operated fractures extended from January 2019 to September 2023. A total of 189 patients were included in the study.

Patients included in the study had extra-articular tibia shaft fractures, which were isolated injuries and treated with operative rigid intramedullary nailing. Additionally, patients with simple malleolar fractures associated with the tibia fracture were also included in the study. The study excluded patients with articular fractures (identified through radiographs or CT scans), open fractures of Gustilo grade 3, evidence of syndesmotic injury, evidence of pathological fractures, pediatric patients, patients treated with alternative fixation modalities, those with insufficient follow-up time to assess postoperative nonunion, and patients lacking immediate operative radiology.

Patients with operative radiographs who were subsequently lost to follow-up were considered united at the normal expected time of union, and subsequent analysis was undertaken with those patients excluded to account for the findings with their inclusion.

The patients underwent operative intervention performed by an orthopedic specialist, with an implant provided by a single manufacturer. The orthopedic surgeons were all at similar levels of training, with at least one specialist supervising cases operated by trainee surgeons.

**Figure 1:**
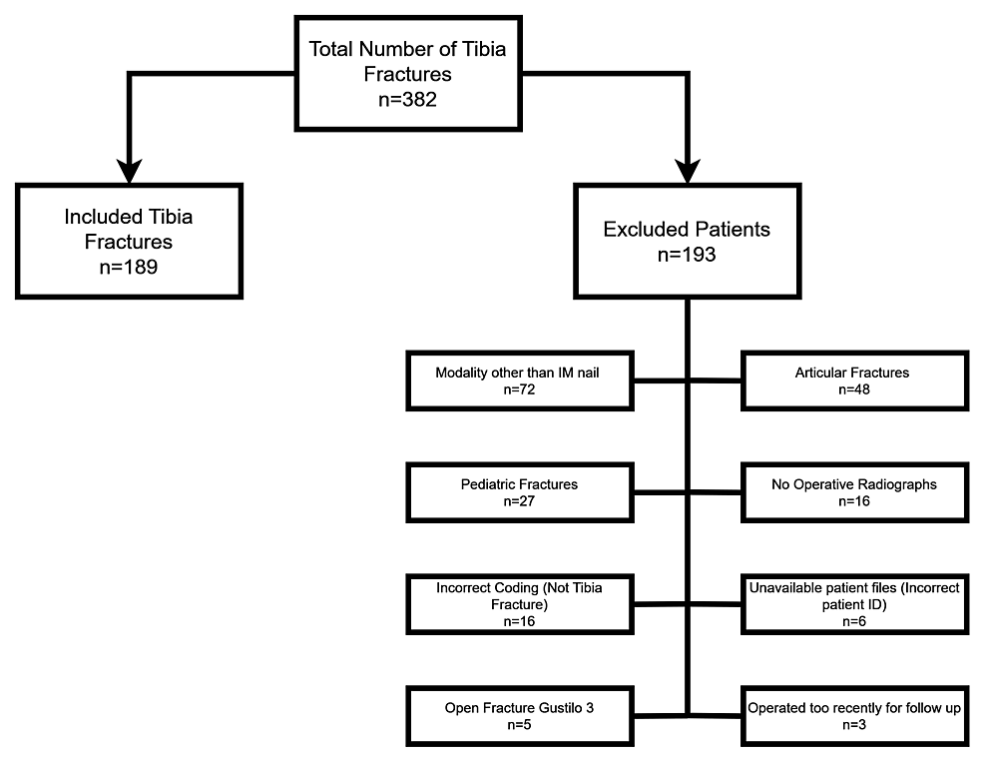
Flowchart detailing exclusion from the study by reason and number. Image credits: Mahmood A. Alam and Ahmed F. Shirazi.

Medical records were searched for every tibia fracture that was treated in the medical center using the surgical electronic logbook. A total of 382 tibia fractures were identified. Of these, 193 patients were excluded for various reasons: 72 underwent a modality other than rigid intramedullary nailing, 48 had articular fractures, 27 had pediatric fractures, 16 lacked operative radiographs, 16 were incorrectly coded (not tibia fractures), 6 had unavailable patient medical records, 5 had open fractures of Gustilo-Anderson grade 3, and 3 were operated on too recently to allow for adequate follow-up. After exclusions, a total of 189 patients were included in the study. A breakdown of excluded patients because of exclusion is illustrated in Figure 1.

The authors collected data retrospectively after agreeing upon a standardized procedure for X-ray assessment and data evaluation. The definition of delayed union was established as the absence of radiological (no callus formation or cortical bridging on standard anterior-posterior [AP] and lateral radiographs) or clinical evidence of union at 16 weeks post-fixation [15-17]. The definition of nonunion was established as the absence of any radiological or clinical evidence of union at 24 weeks and/or a determination by the managing surgeon that the fracture will not progress to union without surgical intervention [5]. Earlier determinations of nonunion were based on the clinical decisions of consultants overseeing the cases. Malunion was defined as a varus/valgus angulation of more than 5° and an AP angulation of more than 10° [18].

Data were analyzed using SPSS version 26 (IBM Corp., Armonk, NY) by an independent statistician. The study received approval from the local ethics committee and the research board of the hospital.

Qualitative variables were analyzed using the chi-square, Fisher’s exact test, and multivariate regression analysis testing. Linear regression analysis was performed for quantitative variables. When qualitative variables were calculated using chi-square and more than 20% of expected values were less than 5, Fisher’s exact test was used where feasible. Where Fisher’s exact test was not feasible, the significance value was not included in the tables to avoid erroneous assumptions.

## Results

Data description and demographics of the dataset

The study included the data of 189 tibia shaft fractures that were operated on with intramedullary nails, irrespective of the presence or pattern of fibula fracture. A total of 160 individuals (84.7%) were male and 29 (15.3%) were female, with a mean age of 40.1 ± 11.9 years.

Of the 189 total patients, 15 (7.9%) sustained a proximal tibia fracture, 61 (32.3%) sustained a midshaft tibia fracture, and 113 (59.8%) sustained distal tibia fractures according to the AO Foundation/Orthopedic Trauma Association (OTA) classification, with articular fractures being excluded. When assessing associated fibular fractures, 41 (21.7%) had proximal fibular fractures, 36 (19%) had midshaft fibular fractures, 82 (43.4%) had distal fibular fractures, and 30 (15.9%) had no associated fibular fracture. The pattern of tibia fractures was transverse (50, 26.5%), oblique (36, 19%), spiral (65, 34.4%), segmental (9, 4.8%), and comminuted in 29 (15.3%) patients. Of particular interest, fractures involving the distal third of both the tibia and fibula, referred to as combined distal tibia and fibula fractures, were observed in 108 patients (57.1%). All other combinations of fracture locations constituted the remaining cases. The mean number of days to operation was short, at 1.7, with a median of one. The average number of days to weight-bearing was 3.1, with a median of zero, indicating immediate weight-bearing (Table 1).

**Table 1:** Frequency and percentage distribution of risk factors (n = 189). IQR, interquartile range

Risk factors	*n* (%)
Gender	
Male	160 (84.7)
Female	29 (15.3)
Age in years (Mean ± SD)	40.1 ± 11.9
Comorbidities	
None	159 (84.1)
One	25 (13.2)
Multiple	5 (2.6)
Level of tibia fracture	
Proximal	15 (7.9)
Midshaft	61 (32.3)
Distal	113 (59.8)
Level of fibular fracture	
Proximal	41 (21.7)
Midshaft	36 (19)
Distal	82 (43.4)
None	30 (15.9)
Pattern of tibia fracture	
Transverse	50 (26.5)
Oblique	36 (19)
Spiral	65 (34.4)
Segmental	9 (4.8)
Comminuted	29 (15.3)
Pattern of fibula fracture	
Transverse	74 (39.2)
Oblique	23 (12.2)
Spiral	35 (18.5)
Segmental	11 (5.8)
Comminuted	16 (8.5)
None	30 (15.9)
Hemoglobin (Mean ± SD)	13.6 ± 1.6
Size of nail in mm	
8.5	22 (11.6)
10	120 (63.5)
11.5	47 (24.9)
Days to operation [Mean, Median (IQR)]	1.7, 1 (1)
Days to weight bearing [Mean, Median (IQR)]	3.1, 0 (0)
Lock of nail	
Static	179 (94.7)
Dynamic	10 (5.3)
Combined subgroup (distal fibula and tibia)	
Other fracture patterns	108 (57.1)
Distal tibia and fibula	81 (42.9)
Combined subgroup (only distal third tibia)	
Other tibial patterns	124 (65.6)
Distal tibia fracture	65 (34.4)
Large or small nail	
Small nail	142 (75.1)
Large nail	47 (24.9)
Combined subgroup (nail size and distal tibia)	
Other fracture patterns	173 (91.5)
Large nail and distal tibia	16 (8.5)

When examining the outcomes, of the total number of patients, 20 (10.6%) required subsequent dynamization, 42 (22.2%) developed nonunion, 40 (21.2%) developed malunion, 55 (29.1%) developed delayed union, and persistent nonunion noted in 10 (5.3%) patients.

The average degrees of malunion was measured to be 10.7°, with a median of 10° and an interquartile range (IQR) of 3. The average time to union was 13.4 weeks, with a median of 8 and an IQR of 12. The majority of patients (159, 84.1%) had no comorbidities, while 25 (13.2%) patients had one comorbidity, and 5 (2.6%) had two or more comorbidities.

Preoperative hemoglobin was 13.6 ± 1.6, and nail sizes were varied, with the majority (120, 63.5%) receiving 10 mm nails, 22 (11.6%) receiving 8.5 mm nails, and 47 (24.9%) receiving 11.5 mm nails.

Most of the nails were statically locked irrespective of fracture pattern or location, with 179 (94.7%) being statically locked at index surgery (Table 2).

**Table 2:** Frequency and percentage distribution of outcomes (n = 189). IQR, interquartile range

Outcomes	*n* (%)
Subsequent dynamization	
Yes	20 (10.6)
No	169 (89.4)
Nonunion (yes/no)	
Yes	42 (22.2)
No	147 (77.8)
Time to nonunion in weeks [Mean, Median (IQR)]	5.1, 0 (0)
Malunion (yes/no)	
Yes	40 (21.2)
No	149 (78.8)
Delayed union (yes/no)	
Yes	55 (29.1)
No	134 (70.9)
Persistent nonunion (yes/no)	
Yes	10 (5.3)
No	179 (94.7)
Degrees of malunion [Mean, Median (IQR)]	10.7, 10 (3)
Complications	
None	99 (52.4)
One	71 (37.6)
Multiple	19 (10.1)
Time to union in weeks [Mean, Median (IQR)]	13.4, 8 (12)

The mean duration of follow-up in the study was 34.6 weeks, with a standard deviation of 42.9 weeks. The mean number of follow-up visits was 3.2, with a standard deviation of 2.8 visits (Table 3).

**Table 3:** Follow-up duration descriptive. IQR, interquartile range; SD, standard deviation

Statistics	Mean ± SD	Median (IQR)
Duration of follow-up in weeks	34.6 ± 42.9	18.0 (42)
Number of follow-up visits	3.2 ± 2.8	3.0 (4)

Risk factors and association with outcomes

Primary analysis showed a significant association between the presence of comorbidities and an increased risk of the need for subsequent dynamization; 8 out of 22 (27%) patients with comorbidities required dynamization, while 12 out of 147 (7.5%) patients with no comorbidities required dynamization (*P* = 0.005). The level of fibula fracture was also significantly associated with an increased risk of nonunion and malunion; fractures associated with distal one-third of fibular fractures developed nonunion in 29 (35%) cases and malunion in 27 (33%) cases, respectively, while other locations had a lower rate of nonunion and malunion (*P* = 0.001, *P* = 0.007) (Table 4).

**Table 4:** Association between risk factors and each of subsequent dynamization, nonunion, and malunion. ^a^Fisher’s exact test. ^b^Chi-square test.

Risk factors	Subsequent dynamization		Nonunion		Malunion	
	Yes	No	Yes	No	Yes	No
	*n* (%)	*n* (%)	*n* (%)	*n* (%)	*n* (%)	*n* (%)
Gender						
Male	12 (7.5)	148 (92.5)	33 (20.6)	127 (79.4)	37 (23.1)	123 (76.9)
Female	8 (27.6)	21 (72.4)	9 (31)	20 (69)	3 (10.3)	26 (89.7)
*P*-value	0.004^a^		0.215^b^		0.121^b^	
Comorbidities						
Yes	8 (26.7)	22 (73.3)	9 (30)	21 (70)	5 (16.7)	25 (83.3)
No	12 (7.5)	147 (92.5)	33 (20.8)	126 (79.2)	35 (22)	124 (78)
*P*-value	0.005^a^		0.264^b^		0.511^b^	
Level of tibia fracture						
Proximal	2 (13.3)	13 (86.7)	2 (13.3)	13 (86.7)	2 (13.3)	13 (86.7)
Midshaft	5 (8.2)	56 (91.8)	18 (29.5)	43 (70.5)	8 (13.1)	53 (86.9)
Distal	13 (11.5)	100 (88.5)	22 (19.5)	91 (80.5)	30 (26.5)	83 (73.5)
*P*-value	0.745^b^		0.217^b^		0.087^b^	
Level of fibular fracture						
Proximal	4 (9.8)	37 (90.2)	3 (7.3)	38 (92.7)	5 (12.2)	36 (87.8)
Midshaft	2 (5.6)	34 (94.4)	6 (16.7)	30 (83.3)	5 (13.9)	31 (86.1)
Distal	13 (15.9)	69 (84.1)	29 (35.4)	53 (64.6)	27 (32.9)	55 (67.1)
None	1 (3.3)	29 (96.7)	4 (13.3)	26 (86.7)	3 (10)	27 (90)
*P*-value	-		0.001^b^		0.007^b^	
Pattern of tibia fracture						
Transverse	6 (12)	44 (88)	13 (26)	37 (74)	10 (20)	40 (80)
Oblique	5 (13.9)	31 (86.1)	6 (16.7)	30 (83.3)	7 (19.4)	29 (80.6)
Spiral	6 (9.2)	59 (90.8)	9 (13.8)	56 (86.2)	12 (18.5)	53 (81.5)
Segmental	0 (0)	9 (100)	3 (33.3)	6 (66.7)	2 (22.2)	7 (77.8)
Comminuted	3 (10.3)	26 (89.7)	11 (37.9)	18 (62.1)	9 (31)	20 (69)
*P*-value	-		0.076^b^		0.720^b^	
Pattern of fibula fracture						
Transverse	9 (12.2)	65 (87.8)	20 (27)	54 (73)	15 (20.3)	59 (79.7)
Oblique	4 (17.4)	19 (82.6)	4 (17.4)	19 (82.6)	4 (17.4)	19 (82.6)
Spiral	5 (14.3)	30 (85.7)	6 (17.1)	29 (82.9)	9 (25.7)	26 (74.3)
Segmental	1 (9.1)	10 (90.9)	3 (27.3)	8 (72.7)	3 (27.3)	8 (72.7)
Comminuted	0 (0)	16 (100)	5 (31.3)	11 (68.8)	6 (37.5)	10 (62.5)
None	1 (3.3)	29 (96.7)	4 (13.3)	26 (86.7)	3 (10)	27 (90)
*P*-value	-		0.534^b^		-	
Size of nail in mm						
8.5	2 (9.1)	20 (90.9)	6 (27.3)	16 (72.7)	7 (31.8)	15 (68.2)
10	12 (10)	108 (90)	28 (23.3)	92 (76.7)	23 (19.2)	97 (80.8)
11.5	6 (12.8)	41 (87.2)	8 (17)	39 (83)	10 (21.3)	37 (78.7)
*P*-value	-		0.564^b^		0.410^b^	
Lock of nail						
Static	20 (11.2)	159 (88.8)	39 (21.8)	140 (78.2)	37 (20.7)	142 (79.3)
Dynamic	0 (0)	10 (100)	3 (30)	7 (70)	3 (30)	7 (70)
*P*-value	0.603^a^		0.695^a^		0.443^a^	
Distal fibula and tibia (combined subgroup)						
Other fracture patterns	10 (9.3)	98 (90.7)	18 (16.7)	90 (83.3)	14 (13)	94 (87)
Distal tibia and fibula	10 (12.3)	71 (87.7)	24 (29.6)	57 (70.4)	26 (32.1)	55 (67.9)
*P*-value	0.495^b^		0.034^b^		0.001^b^	
Only distal third tibia (Combined subgroup)						
Other tibial patterns	14 (11.3)	110 (88.7)	33 (26.6)	91 (73.4)	28 (22.6)	96 (77.4)
Distal tibia fracture	6 (9.2)	59 (90.8)	9 (13.8)	56 (86.2)	12 (18.5)	53 (81.5)
*P*-value	0.662^b^		0.045^b^		0.510^b^	
Large or small nail (combined subgroup)						
Small nail	14 (9.9)	128 (90.1)	34 (23.9)	108 (76.1)	30 (21.1)	112 (78.9)
Large nail	6 (12.8)	41 (87.2)	8 (17)	39 (83)	10 (21.3)	37 (78.7)
*P*-value	0.589^a^		0.322^b^		0.983^b^	
Nail size and distal tibia (combined subgroup)						
Other fracture patterns	17 (9.8)	156 (90.2)	42 (24.3)	131 (75.7)	38 (22)	135 (78)
Large nail and distal tibia	3 (18.8)	13 (81.3)	0 (0)	16 (100)	2 (12.5)	14 (87.5)
*P*-value	0.385^a^		0.025^a^		0.530^a^	
Same versus different levels of fracture						
Same level	13 (12)	95 (88)	28 (25.9)	80 (74.1)	31 (28.7)	77 (71.3)
Different level	7 (8.6)	74 (91.4)	14 (17.3)	67 (82.7)	9 (11.1)	72 (88.9)
*P*-value	0.453^b^		0.157^b^		0.003^b^	
Fibula fixed						
Yes	2 (14.3)	12 (85.7)	6 (42.9)	8 (57.1)	8 (57.1)	6 (42.9)
No	18 (11.5)	139 (88.5)	34 (21.7)	123 (78.3)	31 (19.7)	126 (80.3)
*P*-value	0.670^a^		0.097^a^		0.004^a^	
Open fracture						
Yes	6 (17.1)	29 (82.9)	11 (31.4)	24 (68.6)	10 (28.6)	25 (71.4)
No	14 (9.1)	140 (90.9)	31 (20.1)	123 (79.9)	30 (19.5)	124 (80.5)
*P*-value	0.218^a^		0.147^b^		0.235^b^	

A specific pattern of interest (distal tibia with a concomitant distal fibula fracture) was significantly associated with an increased risk of nonunion and malunion. Fractures of this pattern developed nonunion in 24 (29.6%) cases and malunion in 26 (32.1%) cases, which was significantly higher than other fracture patterns that developed nonunion in 18 (17.6%) cases and malunion in 14 (13%) cases (*P* = 0.034, *P* = 0.001). The patterns of tibia and associated fibula fractures were not associated with nonunion or malunion (*P* = 0.076, *P* = 0.53) (Table 4).

Analysis of a fracture pattern where the fibula and tibia fractures occurred at the same level revealed a significant association with malunion. Those with fractures at the same level developed malunion in 31 cases (28.7%), compared to those with fractures at different levels who developed malunion in 9 cases (11%) (*P* = 0.003). Fixation of fibular fractures was significantly associated with an increased rate of malunion; 8 out of 14 (57%) cases with fibula fixed developed malunion, while 31 out of 126 (19.7%) cases without fibular fixation developed malunion (*P *= 0.004) (Table 4).

The level of tibia and fibula fractures was significantly associated with delayed union; midshaft tibia fractures developed delayed union in 24 (39.3%) cases, and distal fibula fractures developed delayed union in 33 (40%) cases (*P* = 0.028, *P* = 0.004). The level of fibula fracture was also associated with increased overall complications; fractures associated with distal fibula fracture developed complications in 52 (63.4%) cases (*P* = 0.001) (Table 5).

**Table 5:** Association between risk factors and each of delayed union, persistent nonunion, and complications. ^a^Fisher’s exact test. ^b^Chi-square test.

Risk factors	Delayed union		Persistent nonunion		Complications	
	Yes	No	Yes	No	Yes	No
	*n* (%)	*n* (%)	*n* (%)	*n* (%)	*n* (%)	*n* (%)
Gender						
Male	45 (28.1)	115 (71.9)	9 (5.6)	151 (94.4)	75 (46.9)	85 (53.1)
Female	10 (34.5)	19 (65.5)	1 (3.4)	28 (96.6)	15 (51.7)	14 (48.3)
*P*-value	0.488^b^		1.000^a^		0.630^b^	
Comorbidities						
Yes	10 (33.3)	20 (66.7)	2 (6.7)	28 (93.3)	17 (56.7)	13 (43.3)
No	45 (28.3)	114 (71.7)	8 (5)	151 (95)	73 (45.9)	86 (54.1)
*P*-value	0.578^b^		0.661^a^		0.279^b^	
Level of tibia fracture						
Proximal	1 (6.7)	14 (93.3)	1 (6.7)	14 (93.3)	5 (33.3)	10 (66.7)
Midshaft	24 (39.3)	37 (60.7)	4 (6.6)	57 (93.4)	30 (49.2)	31 (50.8)
Distal	30 (26.5)	83 (73.5)	5 (4.4)	108 (95.6)	55 (48.7)	58 (51.3)
*P*-value	0.028^b^		-		0.512^b^	
Level of fibular fracture						
Proximal	5 (12.2)	36 (87.8)	0 (0)	41 (100)	12 (29.3)	29 (70.7)
Midshaft	12 (33.3)	24 (66.7)	3 (8.3)	33 (91.7)	17 (47.2)	19 (52.8)
Distal	33 (40.2)	49 (59.8)	5 (6.1)	77 (93.9)	52 (63.4)	30 (36.6)
None	5 (16.7)	25 (83.3)	2 (6.7)	28 (93.3)	9 (30)	21 (70)
*P*-value	0.004^b^		-		0.001^b^	
Pattern of tibia fracture						
Transverse	18 (36)	32 (64)	3 (6)	47 (94)	26 (52)	24 (48)
Oblique	10 (27.8)	26 (72.2)	1 (2.8)	35 (97.2)	15 (41.7)	21 (58.3)
Spiral	14 (21.5)	51 (78.5)	1 (1.5)	64 (98.5)	23 (35.4)	42 (64.6)
Segmental	2 (22.2)	7 (77.8)	1 (11.1)	8 (88.9)	6 (66.7)	3 (33.3)
Comminuted	11 (37.9)	18 (62.1)	4 (13.8)	25 (86.2)	20 (69)	9 (31)
P-value	0.368^b^		-		0.022^b^	
Pattern of fibula fracture						
Transverse	25 (33.8)	49 (66.2)	5 (6.8)	69 (93.2)	39 (52.7)	35 (47.3)
Oblique	4 (17.4)	19 (82.6)	1 (4.3)	22 (95.7)	9 (39.1)	14 (60.9)
Spiral	9 (25.7)	26 (74.3)	0 (0)	35 (100)	15 (42.9)	20 (57.1)
Segmental	3 (27.3)	8 (72.7)	2 (18.2)	9 (81.8)	7 (63.6)	4 (36.4)
Comminuted	8 (50)	8 (50)	0 (0)	16 (100)	10 (62.5)	6 (37.5)
None	6 (20)	24 (80)	2 (6.7)	28 (93.3)	10 (33.3)	20 (66.7)
*P*-value	0.212^b^		-		0.239^b^	
Size of nail in mm						
8.5	5 (22.7)	17 (77.3)	1 (4.5)	21 (95.5)	11 (50)	11 (50)
10	32 (26.7)	88 (73.3)	8 (6.7)	112 (93.3)	54 (45)	66 (55)
11.5	18 (38.3)	29 (61.7)	1 (2.1)	46 (97.9)	25 (53.2)	22 (46.8)
*P*-value	0.259^b^		-		0.617^b^	
Lock of nail						
Static	50 (27.9)	129 (72.1)	10 (5.6)	169 (94.4)	83 (46.4)	96 (53.6)
Dynamic	5 (50)	5 (50)	0 (0)	10 (100)	7 (70)	3 (30)
*P*-value	0.158^a^		1.000^a^		0.197^a^	
Distal fibula and tibia (combined subgroup)						
Other fracture patterns	26 (24.1)	82 (75.9)	4 (3.7)	104 (96.3)	41 (38)	67 (62)
Distal tibia and fibula	29 (35.8)	52 (64.2)	6 (7.4)	75 (92.6)	49 (60.5)	32 (39.5)
*P*-value	0.079^b^		0.331^a^		0.002^b^	
Only distal third tibia (combined subgroup)						
Other tibial patterns	41 (33.1)	83 (66.9)	9 (7.3)	115 (92.7)	67 (54)	57 (46)
Distal tibia fracture	14 (21.5)	51 (78.5)	1 (1.5)	64 (98.5)	23 (35.4)	42 (64.6)
P-value	0.098^b^		0.168^a^		0.015^b^	
Large or small nail (combined subgroup)						
Small nail	37 (26.1)	105 (73.9)	9 (6.3)	133 (93.7)	65 (45.8)	77 (54.2)
Large nail	18 (38.3)	29 (61.7)	1 (2.1)	46 (97.9)	25 (53.2)	22 (46.8)
*P*-value	0.109^b^		0.456^a^		0.378^b^	
Nail size and distal tibia (combined subgroup)						
Other fracture patterns	53 (30.6)	120 (69.4)	10 (5.8)	163 (94.2)	87 (50.3)	86 (49.7)
Large nail and distal tibia	2 (12.5)	14 (87.5)	0 (0)	16 (100)	3 (18.8)	13 (81.3)
*P*-value	0.158^a^		1.000^a^		0.016^b^	
Same versus different levels of fracture						
Same level	36 (33.3)	72 (66.7)	8 (7.4)	100 (92.6)	62 (57.4)	46 (42.6)
Different level	19 (23.5)	62 (76.5)	2 (2.5)	79 (97.5)	28 (34.6)	53 (65.4)
*P*-value	0.139^b^		0.193^a^		0.002^b^	
Fibula fixed						
Yes	5 (35.7)	9 (64.3)	1 (7.1)	13 (92.9)	10 (71.4)	4 (28.6)
No	45 (28.7)	112 (71.3)	8 (5.1)	149 (94.9)	73 (46.5)	84 (53.5)
*P*-value	0.554^a^		0.545^a^		0.074^b^	
Open fracture						
Yes	9 (25.7)	26 (74.3)	4 (11.4)	31 (88.6)	18 (51.4)	17 (48.6)
No	46 (29.9)	108 (70.1)	6 (3.9)	148 (96.1)	72 (46.8)	82 (53.2)
*P*-value	0.625^b^		0.091^a^		0.617^b^	

Other factors, including open fractures, were not significantly associated with any of the outcome measures of the study (Tables 4-5).

Multivariate regression analysis

Upon subsequent multivariate regression analysis, the presence of distal fibula fractures was found to have increased odds of nonunion (odds ratio [OR] = 4.871, confidence interval [CI] = 1.133-20.948; *P* = 0.033), and delayed union (OR = 3.778, CI = 1.092-13.070; *P* = 0.036). The level of fibula fracture was, however, not significantly associated with malunion or overall complications. The distal level of tibia fracture had increased odds of delayed union (OR = 0.285, *P *= 0.014). No factor was significantly associated with increased odds of malunion or complications.

Days to operation (*P* = 0.022) and hemoglobin levels (*P* = 0.045) were significantly associated with nonunion and not significantly associated with any of the other outcomes (Tables 6-9).

**Table 6:** Binary logistic regression between risk factors and nonunion. CI, confidence interval

Risk factors	*P*-value	Odds ratio	95% CI for odds ratio	
			Lower	Upper
Age (years)	0.450	0.986	0.952	1.022
Level of fibular fracture				
None	Reference			
Proximal	0.428	0.483	0.080	2.926
Midshaft	0.861	1.150	0.239	5.536
Distal	0.033	4.871	1.133	20.948
Pattern of tibia fracture				
Transverse	Reference			
Oblique	0.376	0.582	0.176	1.929
Spiral	0.566	0.727	0.245	2.156
Segmental	0.896	0.889	0.153	5.159
Comminuted	0.829	1.136	0.357	3.620
Hemoglobin	0.045	0.743	0.556	0.994
Days to operation	0.022	1.226	1.030	1.460
Days to weight-bearing	0.309	1.023	0.979	1.070
Combined subgroup (distal fibula and tibia)				
Other fracture patterns	Reference			
Distal tibia and fibula	0.276	0.506	0.149	1.721
Combined subgroup (nail size and distal tibia)				
Other fracture patterns	Reference			
Large nail and distal tibia	0.998	0.000	0.000	-

**Table 7:** Binary logistic regression between risk factors and malunion.

Risk factors	*P*-value	Odds ratio	95% CI for odds ratio	
			Lower	Upper
Age	0.465	1.013	0.978	1.049
Level of tibia fracture				
Midshaft	Reference			
Proximal	0.859	1.279	0.085	19.354
Distal	0.925	0.938	0.246	3.578
Level of fibula fracture				
None	Reference			
Proximal	1.000	-	-	-
Midshaft	1.000	-	-	-
Distal	1.000	-	-	-
Pattern of fibula fracture				
None	Reference			
Transverse	1.000	-	-	-
Oblique	1.000	-	-	-
Spiral	1.000	-	-	-
Segmental	1.000	-	-	-
Comminuted	1.000	-	-	-
Hemoglobin	0.827	1.029	0.794	1.335
Days to operation	0.460	1.086	0.872	1.352
Days to weight-bearing	0.953	0.998	0.950	1.050
Distal fibula and tibia (combined subgroup)				
Other fracture patterns	Reference			
Distal tibia and fibula	0.573	2.068	0.165	25.855
Same versus different levels of fracture				
Same level	Reference			
Different level	0.728	0.688	0.083	5.677
Fibula fixed				
No	Reference			
Yes	0.065	3.207	0.932	11.033

**Table 8:** Binary logistic regression between risk factors and delayed union.

Risk factors	*P*-value	Odds ratio	95% CI for odds ratio	
			Lower	Upper
Age	0.990	1.000	0.970	1.032
Level of tibia fracture				
Midshaft	Reference			
Proximal	0.130	0.180	0.019	1.661
Distal	0.014	0.285	0.105	0.773
Level of fibular fracture				
None	Reference			
Proximal	0.864	0.863	0.161	4.622
Midshaft	0.496	1.661	0.386	7.152
Distal	0.036	3.778	1.092	13.070
Hemoglobin	0.095	0.818	0.646	1.036
Days to operation	0.903	0.990	0.844	1.162
Days to weight-bearing	0.546	1.013	0.972	1.055
Combined subgroup (distal fibula and tibia)				
Other fracture patterns	Reference			
Distal tibia and fibula	0.620	1.362	0.402	4.619

**Table 9:** Binary logistic regression between risk factors and complications.

Risk factors	*P*-value	Odds ratio	95% CI for odds ratio	
			Lower	Upper
Age	0.615	1.008	0.978	1.038
Level of fibula fracture				
None	Reference			
Proximal	0.960	0.966	0.249	3.751
Midshaft	0.354	2.139	0.429	10.673
Distal	0.076	3.035	0.890	10.345
Pattern of tibia fracture				
Transverse	Reference			
Oblique	0.327	0.631	0.251	1.586
Spiral	0.478	0.728	0.302	1.751
Segmental	0.554	1.630	0.323	8.235
Comminuted	0.448	1.519	0.516	4.469
Hemoglobin	0.718	0.960	0.771	1.196
Days to operation	0.082	1.232	0.974	1.558
Days to weight-bearing	0.497	1.015	0.972	1.061
Distal fibula and tibia (combined subgroup)				
Other fracture patterns	Reference			
Distal tibia and fibula	0.666	1.317	0.378	4.587
Nail size and distal tibia (combined subgroup)				
Other fracture patterns	Reference			
Large nail and distal tibia	0.306	0.466	0.108	2.009
Same versus different levels of fracture				
Same level	Reference			
Different level	0.868	0.915	0.318	2.630

Subgroup analysis

The authors conducted a subgroup analysis, excluding those who had no follow-up and were considered united, as outlined in the Materials and Methods section. These comprised 28 (14.8%) patients of the sample size. The authors repeated all the previous statistics after the exclusion. The results were then compared with previous analyses to validate the initial results.

The results of the primary analysis indicated that the majority of significant factors remained statistically significant even after the exclusion. Regression analysis, however, showed that the level of fibula fracture is no longer significantly associated with nonunion (OR = 3.502, *P *= 0.101), while the level of tibia fracture remained significantly associated with delayed union (OR = 0.3, *P *= 0.015). Level of hemoglobin (OR = 0.708, *P* = 0.034) and days to operation (OR = 1.208, *P *= 0.034) remained significantly correlated with nonunion. This may be attributed to the reduction of sample size and further studies are needed to determine this conclusively. The authors included all replicated analyses in the Appendices.

## Discussion

Optimal fracture healing has long been a unique challenge that every orthopedic provider is concerned with. Various fracture patterns have been shown to influence the union rate independent of reduction and stabilization techniques [19]. This study aimed to examine the effect of fracture pattern and location on the rate of nonunion, malunion, and delayed union in tibia fractures treated with intramedullary nailing.

There remains a lack of consensus on the definitions of nonunion and delayed union [20]; various authors advocated different timeframes as suggestive of nonunion, ranging from as early as eight weeks to as late as nine months [13,21]. The significance of nonunion, however, is a point of agreement between orthopedic providers, with various studies demonstrating a significantly deleterious effect on patients' quality of life and a significant increase in healthcare costs [5,6].

While intramedullary nailing has long been established as an effective modality for treating extraarticular tibia fractures [22], our data suggest that it should be performed while paying particular attention to tibial and fibular fracture location. The data suggest that the presence of a distal fibular fracture should raise the attention of the surgeon to the increased likelihood of nonunion; however, further study is needed to validate this finding as this was not observed when excluding patients with no follow-up. Careful operative fixation of the fracture with optimization of reduction and consideration of using an autograft or bone-stimulating agents may be indicated [23,24]. The use of bone-stimulating agents should be balanced in light of their novelty and the controversy surrounding their efficacy [25]. A recent meta-analysis advocated the use of bone-stimulating agents in the acute setting, citing a higher rate of union and a reduced rate of revision surgery. This is a concept that may be difficult for orthopedic surgeons to adapt to initially but could find increasing acceptance as more evidence becomes available [26]. While these novel agents may not yet enjoy the full support of the orthopedic community, they certainly belong in the conversation that aims to tackle complex and serious clinical conditions, including tibia fracture nonunion.

Consideration of dynamic locking is probably prudent in certain fracture patterns that permit it. The data in this study suggest an increase in the need for subsequent dynamization in fractures with associated distal fibular fractures. If the tibia fracture pattern is axially stable, leaving the nail in dynamic or unlocked mode may facilitate healing and decrease the need for subsequent surgery to dynamize the nail [27].

It is also worth noting that the presence of both distal tibia and distal fibula fractures together significantly raises the risk of complications, according to the data explored in this study, which again places significant emphasis on extra careful consideration of methods of fixation in such fractures.

In the center where this study was conducted, it is standard practice to leave the fibula unfixed when using intramedullary nailing for tibial shaft fractures due to concerns about nonunion and varus malunion. It is interesting to note that fibular fixation appeared unrelated to malunion or nonunion in our study, a finding that is of great relevance to practice and may support leaving the fibula unfixed unless clinically indicated for other reasons. This must be opposed to evidence in recent studies that have shown that fixation of the fibula in distal third tibia fractures is not associated with increased complications and may serve to obtain better reduction and augment the tibial fixation [28]. The operating surgeon should exercise his judgment and individualize cases when deciding to fix the fibula in the distal one-third of fractures of the tibia.

The authors acknowledge some limitations of the study. A small proportion of the data collected was for patients who had inconsistent, infrequent, or sporadic follow-up. Patients with good evidence of union on follow-up postoperative X-rays, albeit incomplete radiological union that was subsequently lost to follow-up, were marked as united at the normal expected time of union. Patients with no evidence of union at nine months who had not received surgical intervention were labeled as persistent nonunions. The exclusion of patients with no follow-up reduced the sample size and may have affected the interpretation of initially significant results on primary analysis, the retrospective study design limited access to these excluded patients for further follow-up.

Patients who had poor follow-up are unlikely to be random as most patients who suffer tibia fractures are young expatriate workers in the setting of the study and patients with uneventful postoperative courses are more likely to be lost to follow-up when compared to those with complications. The authors have attempted to run the statistics twice to adjust for this and included subsequent results in the Appendices for reference. There are also confounding factors that cannot be controlled due to the retrospective study design - a limitation that is inherent to this type of study design.

This was a single-center study with a limited sample size, which may affect the interpretation of the data.

## Conclusions

The level and pattern of some tibia and fibula fractures were associated with nonunion, malunion, and delayed union. Orthopedics providers should consider additional fixation of fibula fractures, bone-healing stimulating agents, dynamic locking of nails, and overall meticulous care for alignment and reduction intraoperatively in selected high-risk fracture patterns. Further studies with close and regular follow-up are needed to clarify certain relationships that are nonsignificant after the exclusion of patients with no follow-up. Careful preoperative planning would be advised, particularly for patients with high-risk fracture patterns.

## Appendices

**Table 10:** Association between risk factors and each of subsequent dynamization, nonunion, and malunion (exclusion done). ^a^Fisher’s exact test. ^b^Chi-square test.

Risk factors	Subsequent dynamization		Nonunion		Malunion	
	Yes	No	Yes	No	Yes	No
	*n* (%)	*n* (%)	*n* (%)	*n* (%)	*n* (%)	*n* (%)
Gender						
Male	11 (8.3)	122 (91.7)	33 (24.8)	100 (75.2)	33 (24.8)	100 (75.2)
Female	8 (28.6)	20 (71.4)	9 (32.1)	19 (67.9)	3 (10.7)	25 (89.3)
P-value	0.006^a^		0.422^b^		0.104^b^	
Comorbidities						
Yes	7 (25.9)	20 (74.1)	9 (33.3)	18 (66.7)	5 (18.5)	22 (81.5)
No	12 (9)	122 (91)	33 (24.6)	101 (75.4)	31 (23.1)	103 (76.9)
*P*-value	0.021^a^		0.347^b^		0.599^b^	
Level of tibia fracture						
Proximal	2 (15.4)	11 (84.6)	2 (15.4)	11 (84.6)	2 (15.4)	11 (84.6)
Midshaft	5 (9.4)	48 (90.6)	18 (34)	35 (66)	8 (15.1)	45 (84.9)
Distal	12 (12.6)	83 (87.4)	22 (23.2)	73 (76.8)	26 (27.4)	69 (72.6)
*P*-value	0.775^b^		0.235^b^		0.187^b^	
Level of fibula fracture						
Proximal	4 (10.5)	34 (89.5)	3 (7.9)	35 (92.1)	5 (13.2)	33 (86.8)
Midshaft	2 (6.7)	28 (93.3)	6 (20)	24 (80)	4 (13.3)	26 (86.7)
Distal	12 (16.9)	59 (83.1)	29 (40.8)	42 (59.2)	25 (35.2)	46 (64.8)
None	1 (4.5)	21 (95.5)	4 (18.2)	18 (81.8)	2 (9.1)	20 (90.9)
*P*-value	-		0.001^b^		0.007^b^	
Pattern of tibia fracture						
Transverse	6 (13.6)	38 (86.4)	13 (29.5)	31 (70.5)	9 (20.5)	35 (79.5)
Oblique	4 (13.3)	26 (86.7)	6 (20)	24 (80)	7 (23.3)	23 (76.7)
Spiral	6 (11.3)	47 (88.7)	9 (17)	44 (83)	10 (18.9)	43 (81.1)
Segmental	0 (0)	9 (100)	3 (33.3)	6 (66.7)	2 (22.2)	7 (77.8)
Comminuted	3 (12)	22 (88)	11 (44)	14 (56)	8 (32)	17 (68)
P-value	--------		0.110^b^		0.769^b^	
Pattern of fibula fracture						
Transverse	8 (12.5)	56 (87.5)	20 (31.3)	44 (68.8)	14 (21.9)	50 (78.1)
Oblique	4 (21.1)	15 (78.9)	4 (21.1)	15 (78.9)	3 (15.8)	16 (84.2)
Spiral	5 (16.1)	26 (83.9)	6 (19.4)	25 (80.6)	8 (25.8)	23 (74.2)
Segmental	1 (10)	9 (90)	3 (30)	7 (70)	3 (30)	7 (70)
Comminuted	0 (0)	15 (100)	5 (33.3)	10 (66.7)	6 (40)	9 (60)
None	1 (4.5)	21 (95.5)	4 (18.2)	18 (81.8)	2 (9.1)	20 (90.9)
*P*-value	-		-		-	
Size of nail in mm						
8.5	2 (10.5)	17 (89.5)	6 (31.6)	13 (68.4)	7 (36.8)	12 (63.2)
10	12 (12.4)	85 (87.6)	28 (28.9)	69 (71.1)	19 (19.6)	78 (80.4)
11.5	5 (11.1)	40 (88.9)	8 (17.8)	37 (82.2)	10 (22.2)	35 (77.8)
*P*-value	0.961^b^		0.317^b^		0.256^b^	
Lock of nail						
Static	19 (12.5)	133 (87.5)	39 (25.7)	113 (74.3)	33 (21.7)	119 (78.3)
Dynamic	0 (0)	9 (100)	3 (33.3)	6 (66.7)	3 (33.3)	6 (66.7)
*P*-value	0.600^a^		0.698^a^		0.420^a^	
Distal fibula and tibia						
Other fracture patterns	10 (10.9)	82 (89.1)	18 (19.6)	74 (80.4)	12 (13)	80 (87)
Distal tibia and fibula	9 (13)	60 (87)	24 (34.8)	45 (65.2)	24 (34.8)	45 (65.2)
*P*-value	0.672^b^		0.030^b^		0.001^b^	
Only distal third tibia						
Other tibial patterns	13 (12)	95 (88)	33 (30.6)	75 (69.4)	26 (24.1)	82 (75.9)
Distal tibia fracture	6 (11.3)	47 (88.7)	9 (17)	44 (83)	10 (18.9)	43 (81.1)
*P*-value	0.895^b^		0.065^b^		0.456^b^	
Large or small nail						
Small nail	14 (12.1)	102 (87.9)	34 (29.3)	82 (70.7)	26 (22.4)	90 (77.6)
Large nail	5 (11.1)	40 (88.9)	8 (17.8)	37 (82.2)	10 (22.2)	35 (77.8)
*P*-value	0.866^b^		0.135^b^		0.979^b^	
Nail size and distal tibia						
Other fracture patterns	16 (11)	130 (89)	42 (28.8)	104 (71.2)	34 (23.3)	112 (76.7)
Large nail and distal tibia	3 (20)	12 (80)	0 (0)	15 (100)	2 (13.3)	13 (86.7)
*P*-value	0.390^a^		0.012^a^		0.524^a^	
Same versus different						
Same level	12 (12.8)	82 (87.2)	28 (29.8)	66 (70.2)	29 (30.9)	65 (69.1)
Different level	7 (10.4)	60 (89.6)	14 (20.9)	53 (79.1)	7 (10.4)	60 (89.6)
*P*-value	0.653^b^		0.205^b^		0.002^b^	
Fibula fixed						
Yes	2 (14.3)	12 (85.7)	6 (42.9)	8 (57.1)	8 (57.1)	6 (42.9)
No	17 (12.8)	116 (87.2)	34 (25.6)	99 (74.4)	28 (21.1)	105 (78.9)
*P*-value	1.000^a^		0.207^a^		0.006^a^	
Open fracture						
Yes	6 (22.2)	21 (77.8)	11 (40.7)	16 (59.3)	9 (33.3)	18 (66.7)
No	13 (9.7)	121 (90.3)	31 (23.1)	103 (76.9)	27 (20.1)	107 (79.9)
*P*-value	0.096^a^		0.057^b^		0.134^b^	

**Table 11:** Association between risk factors and each of delayed union, persistent nonunion, and complications (exclusion done). ^a^Fisher’s exact test. ^b^Chi-square test.

Risk factors	Delayed union		Persistent nonunion		Complications	
	Yes	No	Yes	No	Yes	No
	*n* (%)	*n* (%)	*n* (%)	*n* (%)	*n* (%)	*n* (%)
Gender						
Male	44 (33.1)	89 (66.9)	9 (6.8)	124 (93.2)	70 (52.6)	63 (47.4)
Female	10 (35.7)	18 (64.3)	1 (3.6)	27 (96.4)	15 (53.6)	13 (46.4)
*P*-value	0.789^b^		1.000^a^		0.928^b^	
Comorbidities						
Yes	9 (33.3)	18 (66.7)	2 (7.4)	25 (92.6)	16 (59.3)	11 (40.7)
No	45 (33.6)	89 (66.4)	8 (6)	126 (94)	69 (51.5)	65 (48.5)
*P*-value	0.980^b^		0.675^a^		0.461^b^	
Level of tibia fracture						
Proximal	1 (7.7)	12 (92.3)	1 (7.7)	12 (92.3)	5 (38.5)	8 (61.5)
Midshaft	24 (45.3)	29 (54.7)	4 (7.5)	49 (92.5)	30 (56.6)	23 (43.4)
Distal	29 (30.5)	66 (69.5)	5 (5.3)	90 (94.7)	50 (52.6)	45 (47.4)
*P*-value	0.023^b^		-		0.501^b^	
Level of fibula fracture						
Proximal	5 (13.2)	33 (86.8)	0 (0)	38 (100)	12 (31.6)	26 (68.4)
Midshaft	12 (40)	18 (60)	3 (10)	27 (90)	16 (53.3)	14 (46.7)
Distal	32 (45.1)	39 (54.9)	5 (7)	66 (93)	49 (69)	22 (31)
None	5 (22.7)	17 (77.3)	2 (9.1)	20 (90.9)	8 (36.4)	14 (63.6)
*P*-value	0.005^b^		-		0.001^b^	
Pattern of tibia fracture						
Transverse	18 (40.9)	26 (59.1)	3 (6.8)	41 (93.2)	25 (56.8)	19 (43.2)
Oblique	9 (30)	21 (70)	1 (3.3)	29 (96.7)	14 (46.7)	16 (53.3)
Spiral	14 (26.4)	39 (73.6)	1 (1.9)	52 (98.1)	21 (39.6)	32 (60.4)
Segmental	2 (22.2)	7 (77.8)	1 (11.1)	8 (88.9)	6 (66.7)	3 (33.3)
Comminuted	11 (44)	14 (56)	4 (16)	21 (84)	19 (76)	6 (24)
*P*-value	0.381^b^		-		0.032^b^	
Pattern of fibula fracture						
Transverse	24 (37.5)	40 (62.5)	5 (7.8)	59 (92.2)	37 (57.8)	27 (42.2)
Oblique	4 (21.1)	15 (78.9)	1 (5.3)	18 (94.7)	8 (42.1)	11 (57.9)
Spiral	9 (29)	22 (71)	0 (0)	31 (100)	14 (45.2)	17 (54.8)
Segmental	3 (30)	7 (70)	2 (20)	8 (80)	7 (70)	3 (30)
Comminuted	8 (53.3)	7 (46.7)	0 (0)	15 (100)	10 (66.7)	5 (33.3)
None	6 (27.3)	16 (72.7)	2 (9.1)	20 (90.9)	9 (40.9)	13 (59.1)
*P*-value	0.399^b^		-		0.323^b^	
Size of nail in mm						
8.5	5 (26.3)	14 (73.7)	1 (5.3)	18 (94.7)	11 (57.9)	8 (42.1)
10	32 (33)	65 (67)	8 (8.2)	89 (91.8)	50 (51.5)	47 (48.5)
11.5	17 (37.8)	28 (62.2)	1 (2.2)	44 (97.8)	24 (53.3)	21 (46.7)
*P*-value	0.663^b^		-		0.876^b^	
Lock of nail						
Static	49 (32.2)	103 (67.8)	10 (6.6)	142 (93.4)	78 (51.3)	74 (48.7)
Dynamic	5 (55.6)	4 (44.4)	0 (0)	9 (100)	7 (77.8)	2 (22.2)
*P*-value	0.164^a^		1.000^a^		0.174^a^	
Distal fibula and tibia						
Other fracture patterns	26 (28.3)	66 (71.7)	4 (4.3)	88 (95.7)	39 (42.4)	53 (57.6)
Distal tibia and fibula	28 (40.6)	41 (59.4)	6 (8.7)	63 (91.3)	46 (66.7)	23 (33.3)
*P*-value	0.101^b^		0.329^a^		0.002^b^	
Only distal third tibia						
Other tibial patterns	40 (37)	68 (63)	9 (8.3)	99 (91.7)	64 (59.3)	44 (40.7)
Distal tibia fracture	14 (26.4)	39 (73.6)	1 (1.9)	52 (98.1)	21 (39.6)	32 (60.4)
*P*-value	0.180^b^		0.168^a^		0.019^b^	
Large or small nail						
Small nail	37 (31.9)	79 (68.1)	9 (7.8)	107 (92.2)	61 (52.6)	55 (47.4)
Large nail	17 (37.8)	28 (62.2)	1 (2.2)	44 (97.8)	24 (53.3)	21 (46.7)
*P*-value	0.478^b^		0.285^a^		0.932^b^	
Nail size and distal tibia						
Other fracture patterns	52 (35.6)	94 (64.4)	10 (6.8)	136 (93.2)	82 (56.2)	64 (43.8)
Large nail and distal tibia	2 (13.3)	13 (86.7)	0 (0)	15 (100)	3 (20)	12 (80)
*P*-value	0.082^b^		0.600^a^		0.008^b^	
Same versus different						
Same level	35 (37.2)	59 (62.8)	8 (8.5)	86 (91.5)	59 (62.8)	35 (37.2)
Different level	19 (28.4)	48 (71.6)	2 (3)	65 (97)	26 (38.8)	41 (61.2)
*P*-value	0.240^b^		0.196^a^		0.003^b^	
Fibula fixed						
Yes	5 (35.7)	9 (64.3)	1 (7.1)	13 (92.9)	10 (71.4)	4 (28.6)
No	44 (33.1)	89 (66.9)	8 (6)	125 (94)	69 (51.9)	64 (48.1)
*P*-value	1.000^a^		1.000^a^		0.163^b^	
Open fracture						
Yes	9 (33.3)	18 (66.7)	4 (14.8)	23 (85.2)	17 (63)	10 (37)
No	45 (33.6)	89 (66.4)	6 (4.5)	128 (95.5)	68 (50.7)	66 (49.3)
*P*-value	0.980^b^		0.065^a^		0.246^b^	

**Table 12:** Binary logistic regression between risk factors and nonunion (exclusion done). CI, confidence interval

Risk factors	*P*-value	Odds ratio	95% CI for odds ratio	
			Lower	Upper
Age	0.377	0.983	0.948	1.021
Level of fibula fracture				
None	Reference			
Proximal	0.228	0.319	0.050	2.045
Midshaft	0.885	0.885	0.168	4.652
Distal	0.101	3.502	0.782	15.692
Pattern of tibia fracture				
Transverse	Reference			
Oblique	0.459	0.627	0.182	2.157
Spiral	0.653	0.769	0.245	2.412
Segmental	0.627	0.642	0.107	3.844
Comminuted	0.805	1.167	0.341	3.990
Hemoglobin	0.034	0.708	0.514	0.974
Days to operation	0.034	1.208	1.014	1.439
Days to weight bearing	0.183	1.035	0.984	1.090
Distal fibula and tibia				
Other fracture patterns	Reference			
Distal tibia and fibula	0.316	0.519	0.144	1.871
Nail size and distal tibia				
Other fracture patterns	Reference			
Large nail and distal tibia	0.998	0.000	0.000	-

**Table 13:** Binary logistic regression between risk factors and malunion (exclusion done).

Risk factors	*P*-value	Odds ratio	95% CI for odds ratio	
			Lower	Upper
Age	0.746	1.006	0.971	1.042
Level of fibula fracture				
None	Reference			
Proximal	0.463	0.432	0.046	4.057
Midshaft	0.340	0.259	0.016	4.155
Distal	0.722	0.692	0.091	5.267
Hemoglobin	0.751	0.956	0.725	1.261
Days to operation	0.661	1.052	0.838	1.322
Days to weight bearing	0.891	1.004	0.953	1.056
Distal fibula and tibia				
Other fracture patterns	Reference			
Distal tibia and fibula	0.930	1.095	0.146	8.205
Same versus different				
Same level	Reference			
Different level	0.236	0.378	0.076	1.886
Fibula fixed				
No	Reference			
Yes	0.060	3.247	0.953	11.066

**Table 14:** Binary logistic regression between risk factors and delayed union (exclusion done).

Risk factors	*P*-value	Odds ratio	95% CI for odds ratio	
			Lower	Upper
Age	0.729	0.994	0.962	1.027
Level of tibia fracture				
Midshaft	Reference			
Proximal	0.108	0.156	0.016	1.500
Distal	0.015	0.300	0.114	0.789
Level of fibula fracture				
None	Reference			
Proximal	0.434	0.553	0.126	2.435
Midshaft	0.802	1.187	0.310	4.539
Distal	0.079	3.115	0.876	11.084
Hemoglobin	0.073	0.793	0.615	1.022
Days to operation	0.605	0.955	0.801	1.138
Days to weight bearing	0.241	1.029	0.981	1.079

**Table 15:** Binary logistic regression between risk factors and complications (exclusion done).

Risk factors	*P*-value	Odds ratio	95% CI for odds ratio	
			Lower	Upper
Age	0.795	0.996	0.964	1.029
Level of fibula fracture				
None	Reference			
Proximal	0.871	0.883	0.195	4.004
Midshaft	0.404	2.181	0.349	13.635
Distal	0.171	2.581	0.665	10.014
Pattern of tibia fracture				
Transverse	Reference			
Oblique	0.402	0.647	0.234	1.788
Spiral	0.620	0.779	0.290	2.091
Segmental	0.974	1.028	0.193	5.477
Comminuted	0.406	1.680	0.494	5.712
Hemoglobin	0.224	0.854	0.662	1.102
Days to operation	0.129	1.210	0.946	1.547
Days to weight bearing	0.151	1.045	0.984	1.109
Distal fibula and tibia				
Other fracture patterns	Reference			
Distal tibia and fibula	0.583	1.476	0.368	5.918
Nail size and distal tibia				
Other fracture patterns	Reference			
Large nail and distal tibia	0.212	0.381	0.084	1.736
Same versus different				
Same level	Reference			
Different level	0.860	0.901	0.281	2.885

## References

[1] Laurila J, Huttunen TT, Kannus P, Kääriäinen M, Mattila VM (2019). Tibial shaft fractures in Finland between 1997 and 2014. Injury.

[2] Anandasivam NS, Russo GS, Swallow MS (2017). Tibial shaft fracture: a large-scale study defining the injured population and associated injuries. J Clin Orthop Trauma.

[3] Bell A, Templeman D, Weinlein JC (2016). Nonunion of the femur and tibia: an update. Orthop Clin North Am.

[4] McMillan TE, Johnstone AJ (2017). Technical considerations to avoid delayed and non-union. Injury.

[5] Brinker MR, Hanus BD, Sen M, O'Connor DP (2013). The devastating effects of tibial nonunion on health-related quality of life. J Bone Joint Surg Am.

[6] Antonova E, Le TK, Burge R, Mershon J (2013). Tibia shaft fractures: costly burden of nonunions. BMC Musculoskelet Disord.

[7] Patel I, Young J, Washington A, Vaidya R (2022). Malunion of the tibia: a systematic review. Medicina (Kaunas).

[8] Manon J, Detrembleur C, Van de Veyver S, Tribak K, Cornu O, Putineanu D (2019). Predictors of mechanical complications after intramedullary nailing of tibial fractures. Orthop Traumatol Surg Res.

[9] Caubere A, Demoures T, Choufani C, Huynh V, Barbier O (2019). Use of intramedullary nailing in poor sanitary conditions: French Military Medical Service experience. Orthop Traumatol Surg Res.

[10] Kruppa CG, Hoffmann MF, Sietsema DL, Mulder MB, Jones CB (2015). Outcomes after intramedullary nailing of distal tibial fractures. J Orthop Trauma.

[11] Zura R, Braid-Forbes MJ, Jeray K (2017). Bone fracture nonunion rate decreases with increasing age: A prospective inception cohort study. Bone.

[12] Marsh JL, Bonar S, Nepola JV, Decoster TA, Hurwitz SR (1995). Use of an articulated external fixator for fractures of the tibial plafond. J Bone Joint Surg Am.

[13] Tian R, Zheng F, Zhao W, Zhang Y, Yuan J, Zhang B, Li L (2020). Prevalence and influencing factors of nonunion in patients with tibial fracture: systematic review and meta-analysis. J Orthop Surg Res.

[14] Fong K, Truong V, Foote CJ (2013). Predictors of nonunion and reoperation in patients with fractures of the tibia: an observational study. BMC Musculoskelet Disord.

[15] Schofer MD, Block JE, Aigner J, Schmelz A (2010). Improved healing response in delayed unions of the tibia with low-intensity pulsed ultrasound: results of a randomized sham-controlled trial. BMC Musculoskelet Disord.

[16] Romano CL, Romano D, Logoluso N (2009). Low-intensity pulsed ultrasound for the treatment of bone delayed union or nonunion: a review. Ultrasound Med Biol.

[17] Phieffer LS, Goulet JA (2006). Delayed unions of the tibia. J Bone Joint Surg Am.

[18] Vallier HA, Le TT, Bedi A (2008). Radiographic and clinical comparisons of distal tibia shaft fractures (4 to 11 cm proximal to the plafond): plating versus intramedullary nailing. J Orthop Trauma.

[19] Berk T, Halvachizadeh S, Martin DP (2022). Trochanteric fracture pattern is associated with increased risk for nonunion independent of open or closed reduction technique. BMC Geriatr.

[20] Bhandari M, Guyatt GH, Swiontkowski MF, Tornetta P 3rd, Sprague S, Schemitsch EH (2002). A lack of consensus in the assessment of fracture healing among orthopaedic surgeons. J Orthop Trauma.

[21] Obana KK, Lee G, Lee LS (2021). Characteristics, treatments, and outcomes of tibial plateau nonunions: a systematic review. J Clin Orthop Trauma.

[22] Obremskey WT, Cutrera N, Kidd CM (2017). A prospective multi-center study of intramedullary nailing vs casting of stable tibial shaft fractures. J Orthop Traumatol.

[23] Zimmermann G, Wagner C, Schmeckenbecher K, Wentzensen A, Moghaddam A (2009). Treatment of tibial shaft non-unions: bone morphogenetic proteins versus autologous bone graft. Injury.

[24] Fuchs T, Stolberg-Stolberg J, Michel PA, Garcia P, Amler S, Wähnert D, Raschke MJ (2021). Effect of bone morphogenetic protein-2 in the treatment of long bone non-unions. J Clin Med.

[25] Krishnakumar GS, Roffi A, Reale D, Kon E, Filardo G (2017). Clinical application of bone morphogenetic proteins for bone healing: a systematic review. Int Orthop.

[26] Dai J, Li L, Jiang C, Wang C, Chen H, Chai Y (2015). Bone morphogenetic protein for the healing of tibial fracture: a meta-analysis of randomized controlled trials. PLoS One.

[27] Pesciallo CA, Garabano G, Alamino LP, Dainotto TL, Gaggiotti S, Del Sel H (2022). Effectiveness of nail dynamization in delayed union of tibial shaft fractures: relationship between fracture morphology, callus diameter, and union rates. Indian J Orthop.

[28] Kim RG, An VV, Petchell JF (2022). Fibular fixation in mid and distal extra-articular tibia fractures - a systematic review and meta-analysis. Foot Ankle Surg.

